# Variation in a Host–Parasitoid Interaction across Independent Populations

**DOI:** 10.3390/insects3041236

**Published:** 2012-12-05

**Authors:** Saskya van Nouhuys, Suvi Niemikapee, Ilkka Hanski

**Affiliations:** 1 Department of Biosciences, PO Box 65 (Viikinkaari 1), University of Helsinki, FI 00014, Finland; E-Mails: suviniemikapee@gmail.com (S.N.); Ilkka.Hanski@helsinki.fi (I.H.); 2 Department of Ecology and Evolutionary Biology, Cornell University, Ithaca, NY 14853, USA

**Keywords:** *Cotesia melitaearum*, *Melitaea cinxia*, coevolution, encapsulation, immunity, parasitism, susceptibility, virulence

## Abstract

Antagonistic relationships between parasitoids and their insect hosts involve multiple traits and are shaped by their ecological and evolutionary context. The parasitoid wasp *Cotesia melitaearum* and its host butterfly *Melitaea cinxia* occur in several locations around the Baltic sea, with differences in landscape structure, population sizes and the histories of the populations. We compared the virulence of the parasitoid and the susceptibility of the host from five populations in a reciprocal transplant-style experiment using the progeny of five independent host and parasitoid individuals from each population. The host populations showed significant differences in the rate of encapsulation and parasitoid development rate. The parasitoid populations differed in brood size, development rate, pupal size and adult longevity. Some trait differences depended on specific host-parasitoid combinations, but neither species performed systematically better or worse in experiments involving local *versus* non-local populations of the other species. Furthermore, individuals from host populations with the most recent common ancestry did not perform alike, and there was no negative effect due to a history of inbreeding in the parasitoid. The complex pattern of variation in the traits related to the vulnerability of the host and the ability of the parasitoid to exploit the host may reflect multiple functions of the traits that would hinder simple local adaptation.

## 1. Introduction

A parasitoid wasp is a parasite that develops as a larva in or on a host arthropod, eventually killing it. The adult wasp is free living. In parasitoid-arthropod interactions, like host-parasite interactions in general, hosts evolve to avoid or resist parasitism, and parasites evolve to become more virulent. This antagonistic coevolution, which contributes to biological diversity [1], is often expected to lead to systematic and predictable reciprocal changes or local adaptation of one or the other of the partners in the interaction [2,3].

The virulence of a parasitoid and the resistance by the host are the result of a combination of physiological and behavioral mechanisms and are influenced by the environment. An important measure of both host resistance and parasitoid virulence is the ability of a host to kill parasitoid eggs or larvae via mechanisms such as encapsulation. In a given species, this ability may vary geographically [4,5] and depend on a degree of specialization [6,7]. It varies as a response to stress [8,9] and in association with endosymbionts [10,11,12,13,14]. Many traits other than encapsulation can also influence the vulnerability of a host to a parasitoid and the parasitoids’ ability to exploit hosts, including physical [15] and behavioral defenses by the host [16,17], dispersal [18] and diet [19], as well as foraging behavior of the parasitoid [12,20,21], phenological match [22], and life history traits, such as parasitoid clutch size and generation time [23,24]. The significance of particular traits depend on environmental conditions such as weather, landscape structure [25], and the composition of the community with which the species interact. The evolution of relevant traits can depend on shifting direction of selection due to gene-for-gene style coevolution [26,27,28], population history [29], tradeoffs, and genetic constraints such as inbreeding [30].

The mechanisms and patterns of virulence/susceptibility have been explored most thoroughly in three host-parasitoid study systems, in which the hosts are the pea aphid *Acyrthosiphon pisum*, *Drosophila* species, and tropical stem boring moths. Susceptibility of the pea aphid to *Aphidius* parasitoids, and the virulence of the parasitoid vary within and among populations [31,32,33,34]. Resistance by the host is facilitated by symbiotic bacteria, some of which are associated with a toxin-encoding bacteriophage that kills parasitoid eggs. A large body of research on the pea aphid system has elucidated the complex roles of endosymbionts in the variation of aphid susceptibility [14,35,36]. Work on the *Drosophila* system has demonstrated complex variation of susceptibility of the hosts within and among species to parasitoids in the genera *Asobara* [37] and *Leptopilina* [21,28,38]. In this system, the primary mechanism of host resistance is through encapsulation of host eggs [39,40], which is associated with virus-like particles and venom in parasitoids [41,42] and endosymbionts in the hosts [11,12,43]. In the stem-boring moth system, the virulence of the generalist parasitoid *Cotesia sesamiae* and host resistance have been measured in terms of host encapsulation. Encapsulation rate varies geographically [44], with parasitoid and host population [12,45], host species [7], and in association with a *Wolbachia* endosymbiont [12]. In each study system, there is evidence that resistance to parasitoids comes at a cost to hosts [46,47,48] and that increased virulence comes at a cost to parasitoids [46,49,50].

Here, we examine the vulnerability of the butterfly *Melitaea cinxia* (Lepidoptera: Nymphalidae) and the virulence of its specialist parasitoid *Cotesia melitaearum* (Hymenoptera: Braconidae) across five separate localities around the Baltic sea. These localities share the same climate and general plant and insect communities, but they differ in landscape structure, spatial structure and sizes of host and parasitoid populations, and evolutionary history of the species over the past hundreds of years. Using a reciprocal transplant style experiment, we measured the susceptibility of host larvae and the virulence of the parasitoid against parasitoid/host populations originating from their own and other localities. We measured parasitoid brood size, encapsulation rate, pupal mass, rate of development and adult longevity. We ask three specific questions. First, do hosts and/or parasitoids perform better, or worse, when interacting with local versus non-local hosts/parasitoids? Second, the host butterflies from the Åland Islands in Finland, and Uppland on the east coast of Sweden have the most recent common ancestry. Do they perform similarly when interacting with a range of parasitoid populations from different origins? Finally, parasitoids from the tiny island of Pikku Tytärsaari in the middle of the Gulf of Finland have been isolated from conspecific populations for at least 75 years [51]. Do wasps from this small isolated population perform worse, due to strong inbreeding, than their closest relatives from Saaremaa, Estonia, when tested against a range of host populations?

## 2. Material and Methods

### 2.1. The Butterfly and the Parasitoid

The Glanville fritillary, *Melitaea cinxia*, is a Eurasian butterfly that reaches its northern range limit in southern Finland. Adult females lay eggs in clusters of about 150 in June on the host plants *Plantago lanceolata* L. and *Veronica spicata* L. (Plantaginaceae), which grow in dry meadows. The butterfly larvae live gregariously through the summer, diapause gregariously through the winter, resume feeding in the spring, and pupate in the late spring [52]. The parasitoid *Cotesia melitaearum* is entirely specialized on *M. cinxia*, and occurs throughout the geographical range of the butterfly [53]. In the study area, the wasp has two or three generations in each year and per host generation, laying one to 40 eggs in an individual host larva, depending on the host larval instar [54,55].

A host larva is able to encapsulate a fraction of the parasitoid eggs laid in it. Encapsulation ability differs among host families [56] and with the environmental conditions. For instance, it has been shown, using nylon threads as an assay for encapsulation response, that encapsulation ability increases with host food plant quality [8] and with stress [57].

### 2.2. The Study Populations

**Figure 1:**
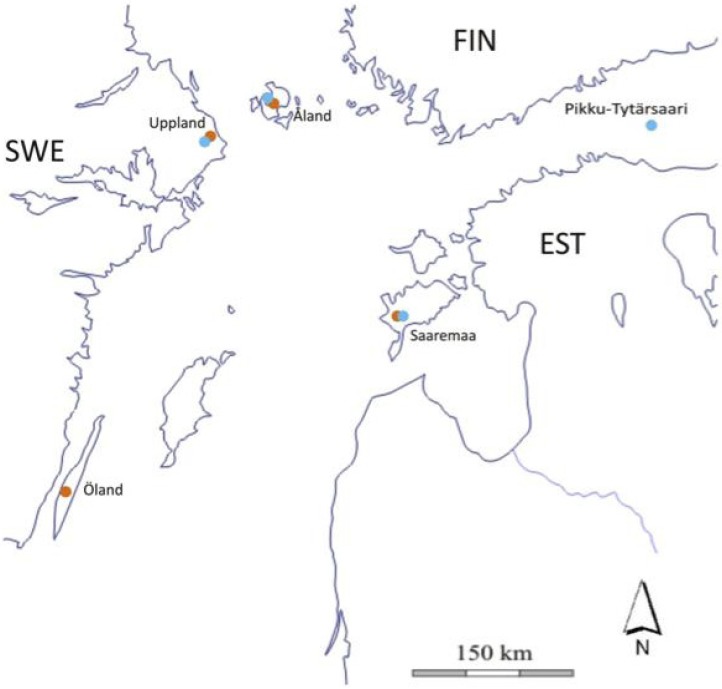
The northern Baltic sea region showing Sweden (SWE), Finland (FI) and Estonia (EST), and the locations of the five study sites. The orange dots show locations from which butterflies were sampled and the blue dots the locations from which parasitoids were sampled.

The insects in this study originate from five northern European localities (Figure 1, Table 1). Those from the Åland Islands (AL) in Finland have been under study for many years. In the Åland Islands, the host occurs as a metapopulation in a fragmented landscape [58]. In any one year, the parasitoid occupies only a small fraction (3 to 10%) of the local butterfly populations, persisting for the most part as small populations constrained by the population dynamics of the host, fragmentation of the landscape, and strongly density-dependent hyperparasitism [54,59]. Isolation of local populations leads to inbreeding in both the butterfly [60,61] and the parasitoid [62]. Uppland (UP), along the eastern coast of Sweden, has a fragmented habitat as in Åland, presumably with similar host and parasitoid population dynamics and degree of inbreeding. The Swedish island of Öland (OL) and the Estonian island of Saaremaa (SA) have large continuous areas of suitable habitat and large butterfly populations. Finally, the tiny island of Pikku Tytärsaari (PT) harbors a single population of the host and of the parasitoid within one coastal meadow of 10 ha. The PT population has been completely isolated for at least 75 years [51]. All the above butterfly populations belong to the same phylogeographic clade (the eastern clade; in Wahlberg and Saccheri [63], the OL population was erroneously assigned to the SE clade), but AL and UP are much more closely related to each other than the others [64]. All the wasppopulations are closely related to populations in western Europe [53]. The isolated population on PT originated from populations that are closely related to SA populations [51]. 

**Table 1:** The host and parasitoid population origins.

	Åland (AL)	Uppland (UP)	Öland (OL) *	Saaremaa (SA)	Pikku Tytärsaari (PT) **
Area (km^2^)	1,480	>1,000	1,342	2,673	2.5
Landscape type	fragmented	fragmented	continuous	continuous	small, isolated
Butterfly inbreeding ^a^	yes	presumed yes	no	no	yes
Wasp inbreeding ^b^	yes	presumed yes	presumed no	no	yes

* only the host, ** only the wasp. ^a^ Saccheri *et al*. 1998, Mattila *et al.* 2012 [51,61], ^b^ Kankare *et al.* 2005 [62].

### 2.3. The Reciprocal Parasitism Experiment

*Melitaea cinxia* larvae were collected from multiple sites within each locality in the autumn 2009. The insects used for the experiment were the progeny of field collected individuals. A fraction of the host larvae collected from all sites except OL were naturally parasitized by the wasp. The parasitoid occurs in OL [53], but by chance was not collected with the sample of host larvae. The host larvae were kept under winter-like conditions in the laboratory until late April 2010, when they were taken out of diapause and reared to adulthood or until parasitoids emerged. Adult butterflies were mated with individuals from the same population but different families. One egg cluster from each of five different mothers from each origin (AL, UP, OL, SA) was used for the experiment. No adult butterflies from the isolated PT island were available. *Cotesia melitaearum* emerged from the field-collected host larvae. In order to obtain the wasps for the experiment, five independent females from each origin (AL, UP, PT, SA) were individually mated with males the same origin but different families. The mated females then each oviposited into laboratory-reared host larvae originating from AL. Fourteen to 18 female progeny from each of these mothers were then mated to unrelated males from the same origin and used in the experiment.

Upon hatching, the host larvae were maintained in a growth chamber in family groups and fed fresh *V. spicata* leaves daily until their third instar, at which point 40 larvae from each of the five families per population were separated into groups of 10. The larvae were reared in groups because they are intrinsically gregarious. One group of 10 larvae from each of the five host families was parasitized by wasps from each of the four wasp populations. Each parasitism, defined as an insertion of the ovipositor, was observed. Three wasps were used to parasitize a set of 10 larvae, each of the three wasps parasitizing three or four host larvae. For every wasp population, this was repeated for each of the five independent host families, The three wasps used for each set of 10 larvae represented different combinations of the progeny of the five original female wasps from each locality. Thus, we replicated five times the interaction between a set of 10 host larvae from each host population with a set of three wasps drawn from the progeny of five female wasps from each wasp population. Notice that the same five host families interacted with all the wasp populations, which makes it possible to analyze the host family effect.

Once parasitized, the host larvae were placed back in the growth chamber and fed *V. spicata* leaves daily. After four days, when parasitoid eggs were hatching, three larvae from each group were dissected and the numbers of live and encapsulated parasitoid eggs and larvae were counted. The remaining host larvae were left to develop until parasitoids emerged, or the host larvae reached diapause. Those that reached diapause without parasitoids emerging from them were dissected, and the numbers of live and encapsulated first, second and third instar parasitoid larvae were counted. The numbers of the third (final) instar parasitoid larvae and pupae were considered together as the individuals emerging before diapause. Many host larvae in the diapause stage contained first or second instar parasitoid larvae, which were designated as destined to overwinter in the host to reach adulthood in the following spring. Host larvae that died without parasitoids coming out of them were dissected and the encapsulated and live parasitoid larvae were counted. Finally, after parasitoid larvae had left the host, the host was dissected and the remaining early instar parasitoid larvae, which would die along with the host, and encapsulated parasitoid larvae were counted.

### 2.4. Brood Size, Pupal Weight and Longevity of C. melitaearum

In the above experiment, the host larvae were dissected at diapause before the pupation of most *C. melitaearum*, and hence the adult size and longevity of the parasitoid could not be measured. To compare the parasitoid populations in these characteristics, we used another set of parasitoids in the following spring to measure brood size at pupation, pupal weight, and adult longevity. To rear this set of wasps, the parent generation from the previous experiment was used to parasitize additional overwintering host larvae from Saaremaa (SA). Twenty-one to 31 host larvae were parasitized by five mothers from each origin. The parasitized hosts were allowed to diapause. Upon breaking diapause, the larvae were reared until wasps pupated. Five females from each family were mated to random males from the same origin and allowed to parasitize five *post* diapause (6th instar) host larvae from Åland (AL). The parasitized larvae were reared under laboratory conditions until the parasitoidsemerged and pupated. The brood size, individual pupal weight at six hours, number of days to adulthood, and sex were recorded. To measure adult longevity, each wasp was kept individually in a temperature-controlled chamber and fed honey water (3:1) daily until death.

### 2.5. Statistical Analyses

The statistical analyses were conducted using JMP v9 (SAS, Cary, NC, USA). In the parasitoid by host origin experiment (Section 2.3), initial and live brood size, fraction of brood encapsulated at four days, rate of development at four days, live brood size at diapause and the fraction maturing in autumn were analyzed with respect the wasp origin, host origin, host family nested within origin, and the interaction between wasp and host origins using least squares ANOVA. Logistic regression was used to analyze the presence/absence of live parasitoids in a host, with respect to the same variables. In the experiment on parasitoid pupae and adult longevity (Section 2.4), least-squares ANOVA was used to evaluate brood size, pupal weight, pupal development time, and adult longevity. Brood size and the interaction of brood size with population origin were included in the model of pupal weight because the latter increases with decreasing brood size due to competition among the parasitoid larvae sharing the same host individual (linear regression R^2^ = 0.08, *p* = 0.024). Pupal weight and the interaction between pupal weight and population origin were included in the model of adult longevity, since the latter tends to increase with wasp size (linear regression R^2^ = 0.13, *p* ≤ 0.0001). Wasp mother was initially included as a factor nested in wasp origin, but the data were unbalanced and as no trend was observed upon inspection, the mother was left out of the final model. For each analysis, the distributions of the variables were tested for normality; no transformations were needed. *Post hoc* independent contrasts were used where needed to compare within multilevel factors.

## 3. Results

### 3.1. Early Parasitoid Development and Encapsulation

**Figure 2:**
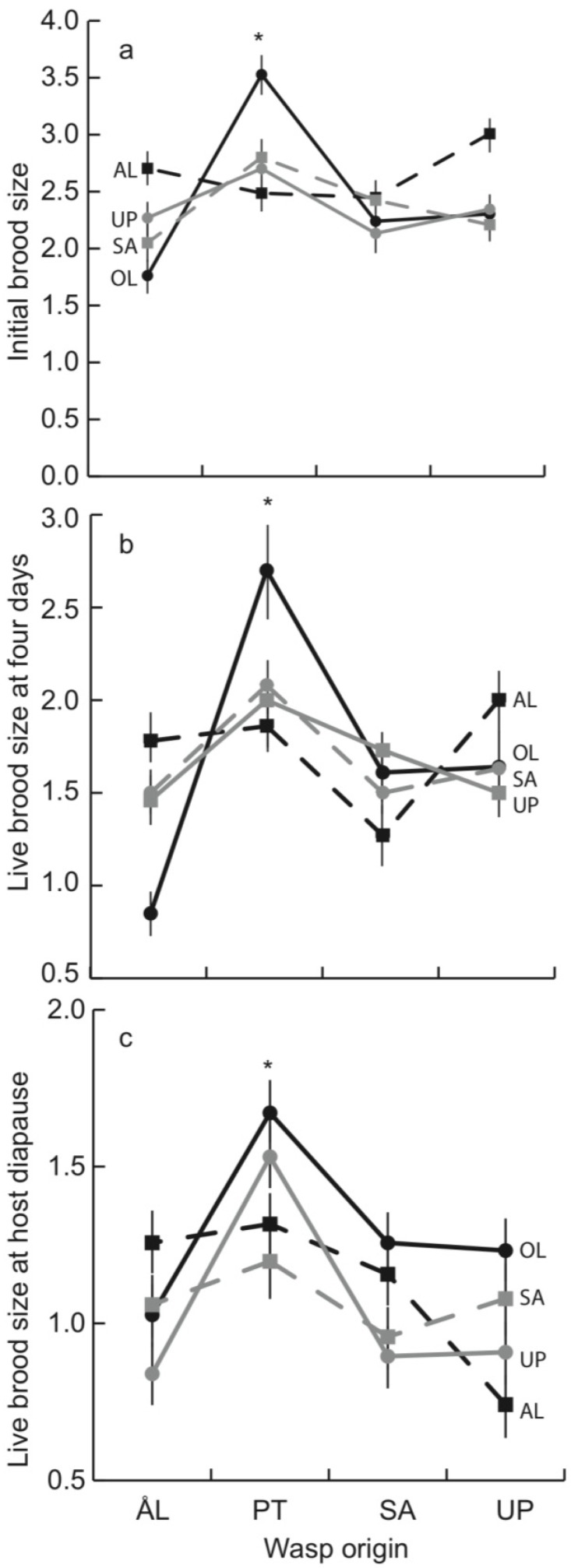
Mean (SE) (**a**) initial (live + dead) brood size (**b**) live brood size at four days, and (**c**) live brood size at host diapause for the parasitoid from each origin developing in the host from each origin; AL (black dashed line), OL (black solid line), SA (gray dashed line) and UP (gray solid line). * indicates that PT wasps differed significantly from other wasp origins in *post hoc* tests, *p <* 0.05.

**Figure 3:**
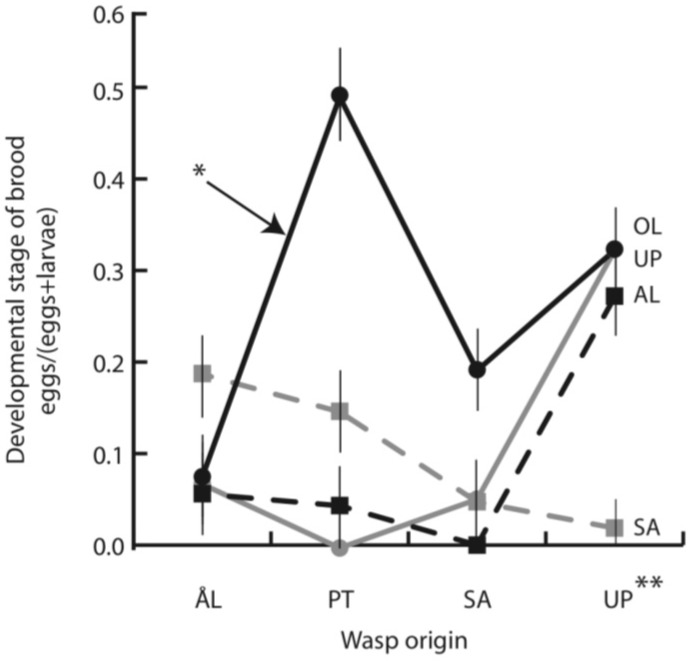
Mean (SE) developmental stage (eggs/(eggs + larvae)) of parasitoid broods from each origin developing in the host larvae from each origin; AL (black dashed line), OL (black solid line), SA (gray dashed line) and UP (gray solid line). * indicates that wasps developed slowly in hosts from OL (independent contrast F_1,382_ = 4.83. *p* = 0.028), ** indicates that wasps from UP developed slowly (independent contrast F_1,173_ = 13.96. *p* < 0.001).

Four days after parasitism most parasitoid eggs had hatched and some had already been encapsulated as eggs or larvae. The initial brood size, including both live and encapsulated individuals, was 2.5 on average, ranging from 0 to 7. There were significant differences among wasp origins (Table 2), with PT wasps producing the largest initial and live broods (Figure 2a,b). Host family had a highly significant effect, but host origin did not (Table 2). The initial brood size of PT wasps was large, primarily due to their performance in OL larvae (Figure 2a), but the overall interaction between wasp and host origins was not quite significant (Table 2). At this early stage, about 30% of parasitoid eggs and larvae were encapsulated with no systematic variation among wasp or host origins (Table 2).The fraction of living parasitoids that were still eggs at day 4 was used as an estimate of the rate of development of the wasps. This fraction ranged from 9 to 25% and varied significantly among the host and wasp origins (Table 2). Wasps from UP and wasps in hosts from OL developed slowly (Figure 3). The rate of development also depended on the specific wasp-host combination (Table 2: significant interaction term). Thirty-five out of the 240 larvae (15%) dissected at four days contained no wasp eggs or larvae. The absence of the wasp did not differ between wasp or host origins nor between host families.

**Table 2:** Analysis of variance of the early parasitoid development.

	Initial brood size					Fraction encapsulated			Fraction eggs		
Source	DF	SS	F	*p*	SS		F	*p*	SS	F	*p*
Model	31	67.95	2.30	<0.001	5.61		1.32	0.132	7.08	3.90	<0.001
Wasp origin	3	13.45	4.70	0.004	0.27		0.66	0.577	0.87	4.69	0.009
Host origin	3	3.25	1.13	0.336	0.17		0.42	0.735	1.05	3.01	0.003
Host family (origin)	16	37.63	2.47	0.002	3.67		1.68	0.054	8.57	2.76	0.002
Host x wasp origin	9	16.49	1.92	0.052	1.27		1.03	0.417	1.84		0.005
Error	173	164.95			23.62				12.84		

### 3.2. Parasitoid Development at Host Diapause

The total parasitoid brood size at host diapause (fifth instar), including both live and encapsulated wasp larvae, was 2.17 on average, which is only slightly smaller than at four days (2.5). Similarly, the same fraction of hosts (16%) as at four days (15%) had no parasitoid progeny in them. However, in clear contrast to early development, at the host diapause stage, a large fraction of the parasitoid individuals in each brood was encapsulated, and the mean live brood size was only 0.91, against 1.75 at four days (Figure 2b,c). At diapause, PT wasps had large live broods. The size of the brood was unrelated to host origin (Table 3, Figure 2c), but it differed between host families (Table 3). The fraction of the brood encapsulated differed among host families and host origins, but was unrelated to wasp origin (Table 3). The largest fraction was encapsulated by UP host and the smallest by OL hosts (UP mean = 0.70(0.04), *post hoc* test F_1,382_ = 4.83. *p* = 0.028; OL mean = 0.52(0.04), *post hoc* test F_1,382_ = 9.08, *p* = 0.029). About 40% of the host larvae that had been parasitized successfully encapsulated the entire brood. Encapsulation of the entire brood differed among host families and host origins but not among wasp origins (Table 4). Host individuals from OL were the least successful in overcoming parasitism (27% encapsulation rate; χ^2^ = 8.76, *p* = 0.003).

**Table 3:** Analysis of variance of parasitoid performance at host diapause.

		Live brood size			Fraction encapsulated			Fraction mature in autumn		
Source	DF	SS	F	*p*	SS	F	*p*	SS	F	*p*
Model	31	88.39	2.45	<0.001	17.09	3.68	<0.001	16.42	4.35	<0.001
Wasp origin	3	12.41	3.55	0.015	0.80	1.78	0.150	4.18	11.46	<0.001
Host origin	3	3.94	1.13	0.337	1.67	3.73	0.011	1.42	3.91	0.009
Host family (origin)	16	64.87	3.48	<0.001	13.00	5.43	<0.001	8.90	4.57	<0.001
Host x wasp origin	9	7.39	0.70	0.704	1.40	1.04	0.405	1.65	1.51	0.143
Error	382	444.91			57.6			46.42		

**Table 4:** Logistic regression analysis of encapsulation of entire parasitoid broods.

	Whole brood encapsulation		
	DF	χ^2^	*p*
Model	31	73.83	<0.001
Wasp origin	3	3.57	0.312
Host origin	3	14.77	0.002
Host family (origin)	16	48.87	<0.001
Host x wasp origin	9	6.8	0.658
Full-reduced log likelihood = 36.92; AICc = 568.21			

**Figure 4:**
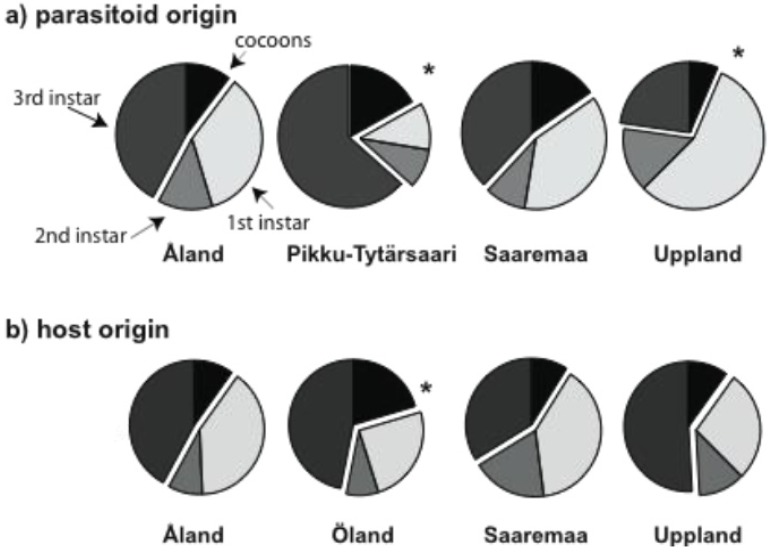
The proportion of the parasitoids in each immature life stage at host diapausestageseparated by parasitoid origin (**a**) and host origin (**b**). In each pie, the fraction maturing in the autumn (two dark pie slices, cocoons and third instar larvae) is offset from the fraction maturing in the spring (two light pie slices, first and second instar larvae). * indicates a statistically significant difference at *p* < 0.05 in the fraction maturing in the autumn.

At the host diapause stage, some *C. melitaearum* larvae develop into their last (third) instar and leave the host, while others remain small (first and second instar) and stay in the host through diapause to become adults in the following spring [55,65]. In this experiment, too, some parasitoid larvae pupated at host diapause, whereas the remaining host larvae were dissected. Those parasitoids that left the host to pupate and those that were in the 3rd instar (though still inside the host) were interpreted as maturing in the autumn. About 35% of all parasitoid larvae matured in the autumn. The fraction of maturing larvae was highest in PT and lowest in UP wasps (Table 3, Figure 4a), and generally, a larger fraction of wasps from all origins matured in OL hosts than in hosts from the other origins (Table 3, Figure 4b).

### 3.3. Brood Size and Adult Longevity in the Spring Generation

**Figure 5:**
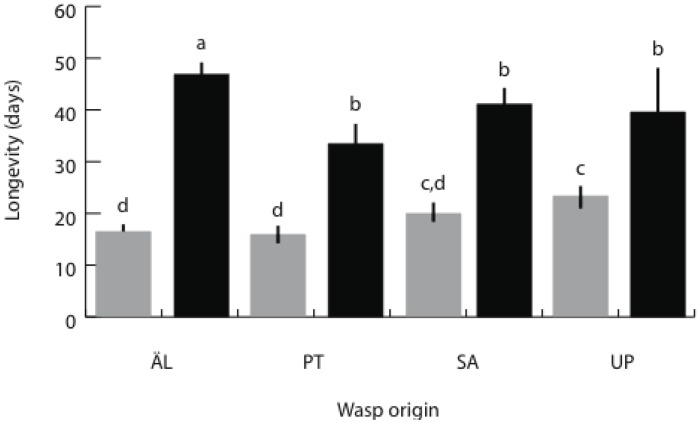
The mean (SE) longevity of the parasitoid from each origin in the spring generation. Males (gray bars) with the same letter on top (c or d) and females (black bars) with the same letter on top (a or b) do not differ significantly in *post hoc* comparisons (*p* < 0.05). See Table 5 for statistical analysis.

Parasitoid brood sizes in the spring generation, the progeny of the overwintering individuals, ranged from 1 to 10 with the mean of 5.1. Wasps from AL had the largest broods (Table 5; post hoc test *F_1,460_* = 24.79, *p* < 0.0001). The wasp pupae took about 6 days to develop into adults, with no differences among populations. Female pupae were larger than males and pupal weights differed among the wasp populations (Table 5), with female AL pupae being the largest (1.77 ± 0.54 mg) (Table 5; *post hoc* test *F_1,456_* = 5.73, *p* = 0.017) and UP males being the smallest (1.19 ± 0.67 mg) (Table 5; *post hoc* test *F_1,456_* = 19.41, *p* < 0.0001). Female wasps lived on average twice as long as males (41 *vs.* 19 days), and AL females lived the longest (Table 5, Figure 5). Adult longevity increased significantly with pupal weight (Table 5).

**Table 5:** Analysis of variance of parasitoid pupal traits and adult performance.

	Pupal brood size				Pupal weight				Adult longevity			
Source	DF	SS	F	P	DF	SS	F	P	DF	SS	F	P
Model	7	363.62	21.98	<0.001	11	19.21	1.75	<0.001	11	713,612.5	62.46	<0.001
Origin	3	300.43	42.36	<0.001	3	2.49	3.63	0.013	3	2,611.66	8.38	<0.001
Sex	1	4.60	1.95	0.164	1	3.69	16.18	<0.001	1	18,138.38	174.64	<0.001
Origin x Sex	3	9.41	1.33	0.265	3	0.55	0.80	0.493	3	2,785.58	8.94	<0.001
Brood size	-	-	-	-	1	3.82	16.73	<0.001	-	-	-	-
Brood size x Origin	-	-	-	-	3	6.44	9.41	<0.001	-	-	-	-
Pupal weight	-	-	-	-	-	-	-	-	1	3,542.55	34.11	<0.001
Pupal weight x Origin	-	-	-	-	-	-	-	-	3	404.96	1.30	0.274
Error	424	1,087			467	104.10			413	49,285.5		

## 4. Discussion

Variation of host and parasitoid life history traits among populations presents a complex pattern of resistance and virulence. There are highly significant differences among origins, but in only a few traits was there significant interaction between the host and parasitoid origins, and there were no systematic differences in the performance of local *versus* non-local hosts and parasitoids. Below, we first discuss specific results related to encapsulation rate and parasitoid life-history traits, and then the broader patterns in terms of genetic differentiation among populations, the potential for local adaptation, and inbreeding.

### 4.1. Encapsulation Rate

Rate of encapsulation is clearly affected by attributes of the host. Four days after parasitism about 30% of the wasp eggs and small larvae had been encapsulated. This fraction was unrelated to host and wasp origin. By the time the host larvae had reached the diapause stage (5th instar), the encapsulation rate had increased to 60%. At this stage the fraction of the brood that was encapsulated depended on host origin but was unrelated to wasp origin. For the parasitoid this result contrasts what has been found for a related species, *Cotesia sesamiae*, which shows variation in encapsulation ability among populations [66]. However, in the latter case, there was partial reproductive isolation between populations using different hosts due to *Wolbachia* infection [12,67]. Population-level differences in the encapsulation ability of the host are not surprising as such differences have been found previously, even in *Cotesia-*Lepidoptera interactions [45], and encapsulation ability of *M. cinxia* is known to vary among families and with stress [8,56].

About 40% of the host larvae successfully encapsulated the entire brood of parasitoids that was laid in them. Thus, a large fraction of the parasitism attempts by the wasp failed, which is surprising because the species is entirely specialized to *M. cinxia* [68]. Host larvae from OL were the least successful at overcoming parasitism, with only 27% of them eliminating the parasitoid by the time they reached the diapause stage.

### 4.2. Parasitoid Brood Size, Pupal Size and Adult Longevity

In the reciprocal parasitism experiment, the summer generation of wasps from PT had the largest initial brood size (number of eggs laid in a host), as well as the largest brood at host diapause. In parasitoid wasps, brood size is often related to female size and fecundity, as well as the ability of the parasitoid to overwhelm the host immune responses [24,69,70]. In parasitoids from PT, however, large broods were not associated with large female body size [71].

Parasitoid broods were larger in the spring generation, when the adult traits were measured, than in the summer generation, when the reciprocal parasitism experiment took place. This is to be expected because, after winter diapause the host larvae are larger than before diapause and, hence, female wasps typically oviposit more eggs into them [72]. In the summer generation PT wasps produced large broods (Figure 3c), whereas in the spring AL wasps produced the largest broods (Table 5). The spring AL wasps also had the heaviest pupae and the longest adult female lifespan. Because of their greater longevity, AL wasps are best adapted in late spring, when they must wait for the next generation of host larvae, which become available about four weeks after the wasps become adult. Great longevity is much less significant in the other two wasp generations, in which host larvae of the same generation are immediately available for parasitism by newly hatched adult parasitoids [55].

### 4.3. Parasitoid Development Rate

Rate of parasitoid development within a host individual is significant both for the parasitoid and the host. Parasitoids that develop slowly experience prolonged exposure to the host immune system, and they are vulnerable to host mortality and hyperparasitism [65]. Slow parasitoid development may be advantageous to the host because it spreads out the resource depletion caused by the parasitoid. On the other hand, for a koinobiont parasitoid such as *Cotesia*, developing slowly may be advantageous as it allows the host to grow, providing greater food resources during wasp larval development [69]. In this study, wasps developed from eggs to first instar slowly in hosts from OL, especially in the case of PT wasps. One explanation for variation in the development rate of gregarious parasitoids is food limitation, which can be related to brood size and host size [73]. In the present case, direct competition among brood mates for food is unlikely when the parasitoids are eggs or first instar larvae. Some other physiological mechanism(s) must play a role in affecting parasitoid development rate in the hosts. It is noteworthy that parasitoid development rate was lowest in OL hosts, which were least able to encapsulate the entire parasitoid brood, suggesting that slow parasitoid development does not necessarily lead to effective host defenses.

When the host larva reaches the diapause stage, the parasitoid larva can stay in the first or second instar and go into diapause, or develop to the third instar, exit the host, become an adult in the autumn, and parasitize nearby host larvae. Normally, a fraction of the progeny of summer wasps become adults in August to produce an additional autumn generation, while the majority stay within the host over the winter [55]. In this experiment about 35% of the wasps matured in the autumn. The fraction and number of wasps maturing in the autumn was highest in PT and lowest in UP hosts. The decision to stay within the host may be affected by resource availability, such that parasitoid larvae within small hosts choose to stay over the winter and finish development when the hosts grow larger in the spring. If this were the case, one might expect larger broods to stay within the host longer. Instead, we found that PT wasps, which had the largest broods, also matured most frequently in the autumn. For any parasitoid it would be advantageous to have three rather than two generations per host generation. This is especially relevant for *C. melitaearum*, which parasitizes only a small fraction of the hosts [55] that have a high rate of overwintering mortality, often exceeding 50% [60,74].

Considering the different host populations, the largest fraction of wasps from all origins matured in the autumn in OL hosts. The high rate of parasitoid population growth due to three rather than two generations per host generation makes the OL host population vulnerable to high rates of parasitism in the spring, Combined with the low rate of encapsulation of entire parasitoid broods by OL host larvae, this makes them appear to be particularly vulnerable to parasitism, potentially leading to population decline [54,55].

### 4.4. Variation in Host Resistance and Parasitoid Virulence

The conventional view of hosts and parasites is that, under a wide range of conditions, host populations evolve to become more resistant to local parasites, and the latter become more virulent, at least up to a point, against local hosts [3]. In the coevolutionary context, one species or the other may be ahead in the “arms race” and there may be temporal fluctuation in host resistance and parasite virulence [75]. Nonetheless, some systematic variation in resistance and virulence traits is expected when comparing local *versus* non-local interactions [2,76]. Local adaptation of parasites to hosts, and hosts to their parasites, has been detected in many organisms [76], but only a few studies have been conducted on local adaptation in parasitoid wasps and their hosts. Henter and Via [31,32] demonstrated the potential for coevolution by quantifying genetic variation of both resistance by the pea aphid *Acyrthosiphon pisum* and of virulence of its parasitoid *Aphidius ervi* within a single pea aphid population. Sandrock [77] found genetic variation in resistance within each of four populations of another aphid, *Aphis fabae*, and virulence of its parasitoid, *Lysiphlebus fabarum*, but no genetic differentiation or genotype specific interactions, suggesting lack of local adaptation in spite of genetic variation. Dupas and colleagues have shown, using *Drosophila* and the parasitoid *L. boulardi*, surprisingly simple gene-for-gene style coevolution of encapsulation between the fly and the parasitoid [50,78]. In contrast, Kraaijeveld and colleagues compared susceptibility of *D. melanogaster* and virulence of another parasitoid, *A. tabida*, across Europe and found no local adaptation. Instead, host resistance was highest in south-central Europe and lower elsewhere, while virulence of the parasitoid showed a cline from low in northern Europe to high in southern Europe. They attributed the lack of local adaption in virulence and resistance to the costs of defense and resistance [5] relative to selection on other traits associated with local environments, and the composition of the communities in which the species occurred [79]. 

In the present study, we found that neither the host nor the parasitoid performed better or worse when interacting with local *versus* non-local hosts/parasitoids. Rather than exhibiting a pattern of local adaptation, host populations differed in their susceptibility to parasitism by wasps from all origins as measured by their ability to encapsulate parasitoid eggs and larvae. Thus UP hosts were able to encapsulate the largest fraction of parasitoid broods and OL hosts were the least able in this respect. In the latter, the result was a low rate of complete recovery from parasitism (encapsulation of all parasitoid eggs and larvae). These results appear to be in line with what has been found in the *Drosophila*-*A. tabida* system and in the pea aphid system, suggesting complex patterns of natural selection on quantitative traits. They differ from what has been found for the parasitoid *C. sesamiae* and its stem-boring moth hosts in Africa. While reciprocal local adaptation has not been tested explicitly, the parasitoid, which has a wide host range, was more virulent to local than novel host populations [80] and host species [81]. Each population in our study was represented by the progeny of only five mothers, therefore we could have missed sampling adapted alleles within the populations by chance. If this were the case, then a larger scale study could reveal local adaptation such as Chinwada [78] found, of relatively simple traits such as encapsulation ability [28].

All of the populations in this study area have relatively recent common ancestry. In the absence of strong selection, populations of recently shared ancestry should have similar characteristics. Among the host populations in this experiment, host butterfly populations from AL and UP have the most recent common ancestry, as assessed by genome-wide genetic variation [64]. Thus, we might expect these two populations to perform similarly when interacting with parasitoid populations from different origins. Though parasitoids’ development rate was similar in these two host populations (Figure 3), they were not particularly similar in other traits. This result indicates that, while there was not local adaptation, there was local genetic differentiation on a short-term scale, on the order of hundreds of generations at most, in traits related to interaction with the parasitoid. 

Another specific comparison among populations in the study can be made between parasitoids from the populations of PT and SA. Those from the small island of Pikku Tytärsaari (PT) have been isolated from other populations for at least 75 years [51]. The colonizers originated from populations that are closely related to populations in Saaremaa (SA) among our study populations, and have subsequently experienced strong inbreeding. Inbreeding depression typically lowers the ability of a population to withstand environmental stress [82], and is a well-established phenomenon in insects, including the host butterfly *M. cinxia* in this study, both in PT [51] and in local populations in AL [61]. We expected that inbreeding would have a negative effect on PT parasitoids, but we found no such evidence, as the PT wasps had the highest fecundity and developed faster than wasps from any other origin. In particular, they did not perform worse than the SA wasp population in any measure. Inbreeding depression in parasitoids is apparently rare, at least in part because of haplodiploidy [30]. In Hymenoptera, males are haploid and, hence, recessive deleterious mutations are expressed and selected against in all populations. Though other potential negative effects of inbreeding apart from the expression of recessive mutations exist [83], purging of deleterious alleles must have a strong effect. In spite of this, it is interesting to note that that there was no evidence of inbreeding depression of encapsulation ability, which is a trait expressed only by females, as it is regulated by factors such as polyDNA virus injected into the host upon parasitism.

## 5. Conclusions

Evolution due to antagonistic interactions between parasites and their hosts generates diversity through local adaptation of one or the other species, and coevolution of both species [1]. This is clearly illustrated in many organisms, such as bacteria [84] and plants and their pathogens [85], where the genetic architecture of virulence and resistance is often simple, and where the traits are significantly modified by small genetic changes that may not affect other fitness components. Parasitoids and their host arthropods are models for ecological and evolutionary research on more complex interactions. A parasitoid and its host live in a complex environment and exert strong selection on one another. But wasp virulence and host resistance are not simple traits and, hence, they are unlikely to evolve independently or quickly. The present study contributes to a very small number of host-parasitoid studies in addressing both host resistance and parasitoid virulence across multiple populations. To our knowledge, this is the only study with a Lepidoptera host, though, otherwise, there is extensive literature on Lepidoptera and their parasitoids [86]. As in the studies on the pea aphid and its parasitoids [31,32], and in *Drosophila* and *Asobara* [5], we found ample variation of traits related to virulence and resistance, but no evidence of local adaptation. Lack of local adaptation most likely reflects the complexity of the traits involved in the interaction. As our understanding of the actual mechanisms that are involved increases, including the genetic basis of virulence and resistance [87] and interactions with symbionts [10,88], we will be better able to decipher the causes of variation of parasitoid virulence and host resistance.
